# Fasciolosis—An Increasing Challenge in the Sheep Industry

**DOI:** 10.3390/ani12121491

**Published:** 2022-06-08

**Authors:** Snorre Stuen, Cecilie Ersdal

**Affiliations:** Department of Production Animal Clinical Sciences, Norwegian University of Life Sciences, N-4325 Sandnes, Norway; cecilie.ersdal@nmbu.no

**Keywords:** *Fasciola hepatica*, sheep, clinical signs, treatment, pathology, histology

## Abstract

**Simple Summary:**

Fasciolosis caused by *Fasciola hepatica* is a serious disease and a huge challenge in the sheep industry. The disease has several clinical manifestations including acute death, anemia, ill-thrift and loss of body condition. Climate change with milder temperatures and heavier rainfall will increase the risk of fasciolosis. Grazing management and treatment with flukicide are at present the only options to restrain *F. hepatica* infection. However, control possibilities are challenging, and resistance to flukicide drugs is increasing. Diagnostic improvements, targeted treatment and vaccines will hopefully increase animal health and welfare on fluke infested pastures in the future.

**Abstract:**

The liver fluke *Fasciola hepatica* may cause severe infection in several mammalian species, including sheep and humans. Fasciolosis is a parasitic disease occurring worldwide in temperate climates and involves intermediate lymnaeid snails as vectors, in Europe the pond snail *Galba truncatula* in particular. In the sheep industry, the disease is a serious welfare and health problem. Fasciolosis is usually classified as acute, subacute or chronic according to the number and stage of flukes present in the liver, but with a considerable overlap. Acute disease, associated with a large number of migrating larvae, often results in sudden death due to acute and massive hemorrhage, while chronic fasciolosis is characterized by anemia, hypoalbuminaemia and weight loss. The management of fasciolosis is an increasing challenge in the sheep industry. Early diagnostic tests are limited. Protective immunity against liver flukes in sheep is low or lacking, and vaccines are not yet available. Treatment and control possibilities are challenging, and resistance to flukicide drugs is increasing. In addition, climate change with warmer and more humid weather will have a substantial effect on the establishment of both flukes and snails and will most likely increase the future distribution of *F. hepatica*.

## 1. Introduction

Fasciolosis is a worldwide disease in several mammalian species, caused by liver flukes in the genus *Fasciola,* namely *F. hepatica* and *F. gigantica.* The two species differ in size, but hybrids may occur forming intermediate forms in areas where the two species overlap [1]. It has earlier been estimated that around 550 million cattle and sheep are infected with *F. hepatica* worldwide [2]. Fasciolosis causes a huge financial loss especially to farmers, but also to butchers and consumers. The infection is considered a major public health problem, and it is estimated that between 50–100 million humans may be infected via contaminated water and vegetables, mostly due to ingestion of watercress (*Nasturtium officinale*) [1,3,4]. In the present study we will only focus on *F. hepatica* infection in sheep.

Liver fluke infection affects both productivity and welfare, and represents a considerable economic burden on livestock farming, with subclinical losses contributing a large proportion of the cost, including reduced milk yield, fertility and growth rates [5,6]. Fasciolosis in sheep has also been associated with a variety of conditions including black disease (infection by *Clostridium novyi*), parasitic gastroenteritis and metabolic diseases, especially around lambing time, such as hypocalcaemia and pregnancy toxaemia [7]. It is also a predisposing risk factor for mastitis [8], and inflammatory mediators from liver damage may affect early pregnancy [9].

## 2. Life Cycle

The life cycle involves different developmental stages in the following order: egg–miracidium–sporocysts–rediae–cercariae–metacercariae–juveniles–adults. One single fluke may produce around 25,000 eggs per day [10]. At temperatures between 0–10 °C, the eggs may remain viable for at least two years, but are killed if exposed to temperatures below −5 °C for more than two weeks [11]. The miracidium stage develops in the eggs at temperatures above 10 °C. Development time is around 6 weeks at 15 °C and 10 days at 22 °C. Light is essential for hatching, while the eggs must be free from feces and completely covered with a film of moisture before further development. After hatching, the miracidium moves by cilia and seeks to find a snail. Indirect hosts of *Fasciola* are freshwater snails of the family *Lymnaeidae*, of which the pond snail *Galba truncatula* seems to be the main host in Europe [1]. Although *G. truncatula* is normally dependent on water, it can survive periods of drought [11]. Permanent habitats include banks of ditches, streams and the edges of small ponds. However, following heavy rainfall or flooding, temporary habitats may include wheel ruts and rain ponds [7].

In the snail, the miracidium develops into sporocysts, later to rediae, and then to the final stage, the cercariae. More than 600 cercariae develop from each miracidium, and about five weeks after having entered the snail, the cercariae may be shed if the temperature and moisture are favourable. Cercariae swim to the herbage on which they attach, lose their tails, secrete a tough cyst wall and become metacercariae. Under optimal conditions, they can survive for a year, but many are lost due to desiccation and freezing [11].

Once ingested by the sheep, the metacercariae excyst in the duodenum and release juveniles that penetrate the intestinal mucosa and traverse the peritoneal cavity to reach the liver capsule, which they penetrate. They migrate through the liver parenchyma to end up in the bile ducts, where they mature to become adult flukes and are then leaf-shaped and approximately 2.5 cm long. The flukes are hermaphrodites, and egg-laying starts about 10–12 weeks after the initial infection. In the bile ducts, they can live for many years.

Infection mainly occurs on pasture, but infection through ingestion of silage may occur, although proper anaerobic fermentation results in loss of viability of metacercariae within two weeks, while they may remain viable for up to 10 weeks in spoiled silage [12]. In addition, experimental studies indicate that metacercariae may remain viable for 50 days within dry hay, although contaminated and poorly stored hay may contain *F. hepatica* for several months [13].

At least in north-western Europe, infection in ruminants can be split into two major infection peaks, “winter” infection and “summer” infection [14]. Summer infection is characterized by the infection of snails in May/June, with production of metacercariae in August/September, while in the case of winter infection, snails are infected in late autumn. In the latter case, the infection overwinters in the snails and metacercariae are already available from May to July. Summer infection seems to be the dominant infection pattern in northern Europe [15], but according to climatic models, there will be an increase in winter infection in the future [16]. However, a considerable infection overlap may occur in different areas, especially with a long grazing period. The occurrence and frequency of infection will also depend on local environmental factors, such as snail habitat availability and grazing management [15].

## 3. Pathogenesis

The juvenile and adult flukes have an outer surface called the tegument that protects the fluke from enzymes and immune attacks, but also suppresses the host’s immune system [17]. The flukes mechanically damage the liver tissue while moving, in addition to feeding on the tissue. The regenerative capacity of the liver is high and will be initiated by the moving parasites, and this can be seen from an influx of mononuclear cells and eosinophils and subsequent fibrosis. The rapid movement of the flukes is a way to escape the immune system. In the acute phase, *F. hepatica* will mount a mixed cellular (Th1) and humoral (Th2) immune response. When the infection progresses, the Th2 response will increase and the Th1 response will decrease. In addition, the flukes release immunomodulatory molecules to facilitate survival both in the acute and the chronic stage of the infection [18]. This also increases susceptibility for bacterial infections. When the flukes end up in the bile ducts, they start to feed on blood, bile, lymph and tissue, and anemia and hypoproteinaemia develop. The tegument has spines that can be used for puncturing blood vessels [17]. Sheep do not develop protective immunity against *F. hepatica* [11].

## 4. Clinical Signs

Fasciolosis is usually classified as acute, subacute or chronic related to clinical appearance, mainly correlated to the number and stage of flukes present in the liver, although with a considerable overlap.

Acute disease, associated with larval migration of a large number of juveniles over a short period of time, often results in sudden death due to acute and massive hemorrhages. However, trickle infection over weeks or months is not uncommon. Acute and chronic infection may therefore occur simultaneously [19]. Examination of other sheep in the same flock may show lethargic individuals, with pale mucosa, and exhibiting dyspnea. Rough handling can cause liver rupture and subsequent death. Differential diagnoses may include haemonchosis, clostridial infections and acute “pasteurellosis” [7].

In the subacute phase, there is a gradual loss of body condition and infected animals may show ill-thrift. Examination of affected animals will demonstrate pale mucosae. Blood samples will reveal a hypochromic macrocytic anemia, low packed cell volume, hypoalbuminemia, and elevated liver enzymes (AST, GGT, GLDH). Death may also occur. At necropsy, a large number of immature flukes is detected in the liver parenchyma, although adult flukes may also be present in the major bile ducts. In the autumn, many lambs can be slaughtered with subacute fasciolosis that exhibit rather extensive changes in the liver, but without clinical signs.

Chronic fasciolosis is the most common manifestation of the disease complex. Progressive loss of the condition is typical. Anemia can be severe, and the mucosae are often extremely pale. Death may occur especially in late pregnancy, although many sheep show no more than ill-thrift if the fluke burden is low. In addition, submandibular oedema is typical for the chronic form, and fluke eggs will be present in feces. Chronic fasciolosis is also characterized by hypoalbuminemia, eosinophilia and elevated liver enzymes. Differential diagnoses are haemonchosis, malnutrition, cobalt deficiency and other conditions related to ill-thrift, such as Johne’s disease and scrapie [20].

## 5. Gross Pathology

**Figure 1:**
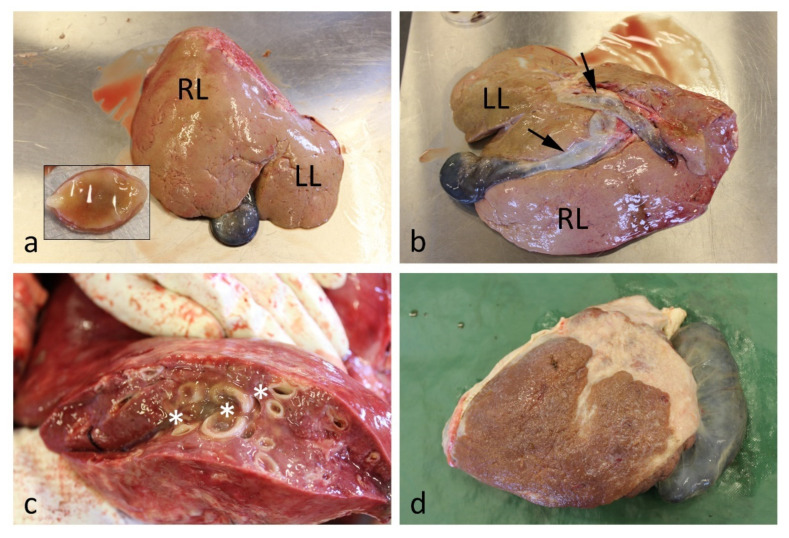
Gross pathology of chronic fasciolosis. (**a**). The diaphragmatic surface of the liver is irregular with elongated shallow depressions on both the left lobe (LL) and right lobe (RL). The inset image shows a mature *Fasciola hepatica*. (**b**). On the visceral side, the cystic bile duct that empties into the gall bladder and the duct in the hilar area (arrows) is markedly dilated. The same rough surface as in image (**a**) is evident. (**c**). The cut surface shows many dilated bile ducts with thickened walls (asterisks). The liver parenchyma is marbled because of fibrosis. (**d**). A severely fibrotic, small, and deformed liver with a dilated gall bladder.

Early changes affect the liver capsule and surface, especially the left diaphragmatic surface [21]. There can be fibrin and thread-like hemorrhagic areas on the surface after the invasion of juvenile flukes. These young flukes will migrate through the parenchyma. On the cut surface, there may be focal to multi-focal pale necrotic areas and bleedings in the sub-acute stage, and juvenile flukes can be found in the liver parenchyma. If the invasion is massive, larger and fatal hemorrhages can occur. In such cases, the mucous membranes, carcass and organs will be pale. There can be large blood clots associated with ruptures in the liver, and free blood in the abdomen. Later in the subacute stage, liver changes may vary from focal thread-like pale migratory patterns, including fibrous tags on the surface, to rather extensive changes affecting both the parietal and visceral surfaces. The left hepatic lobe is usually more affected than the right. In the chronic stage, there are fibrotic scars, visible as thin and elongated shallow depressions on the surface, matching the subacute changes (Figure 1a,b). The bile ducts are dilated, and the duct walls are thickened [22]. This is especially evident for the cystic duct that empties into the neck of the gallbladder and the ducts in the hilar area (Figure 1b). Larger, dilated bile ducts may also protrude on the visceral surface (Figure 1c). On the cut surface, dilated ducts with thickened wall are easily visible (Figure 1d). Mature flukes will be found in the bile ducts and in the gallbladder. The hepatic tissue consistency will be increased because of fibrosis in the triads. A low number of flukes can be encysted in the parenchyma. The cysts are most often found at the visceral surface, where they can undergo a process of caseous necrosis and mineralization [23]. In severe and advanced chronic cases, there can be extensive fibrotic areas and a loss of hepatic tissue (Figure 1d).

At necropsy of chronic cases, a pale, anemic carcass is evident. The body condition is usually poor or the animal can be cachectic. There is often subcutaneous oedema that can be generalized or concentrated to the ventral part of the head and neck (bottle jaw). Serous fluid is commonly found in the abdomen, thorax and pericardial sack as a consequence of hypoproteinaemia. The lungs are pale, presenting with a wet cut surface and fluid and foam in the airways. Acute death after treatment may occur, most probably due to a massive destruction of flukes followed by the release of toxic compounds, although the main cause is unknown. However, this fatal outcome can reveal the same chronic changes as described above, but without findings of flukes. Sometimes, flukes can enter hepatic veins by accident and end up in unusual sites such as the lungs and uterus, causing abscessation [23].

## 6. Histopathology

**Figure 2:**
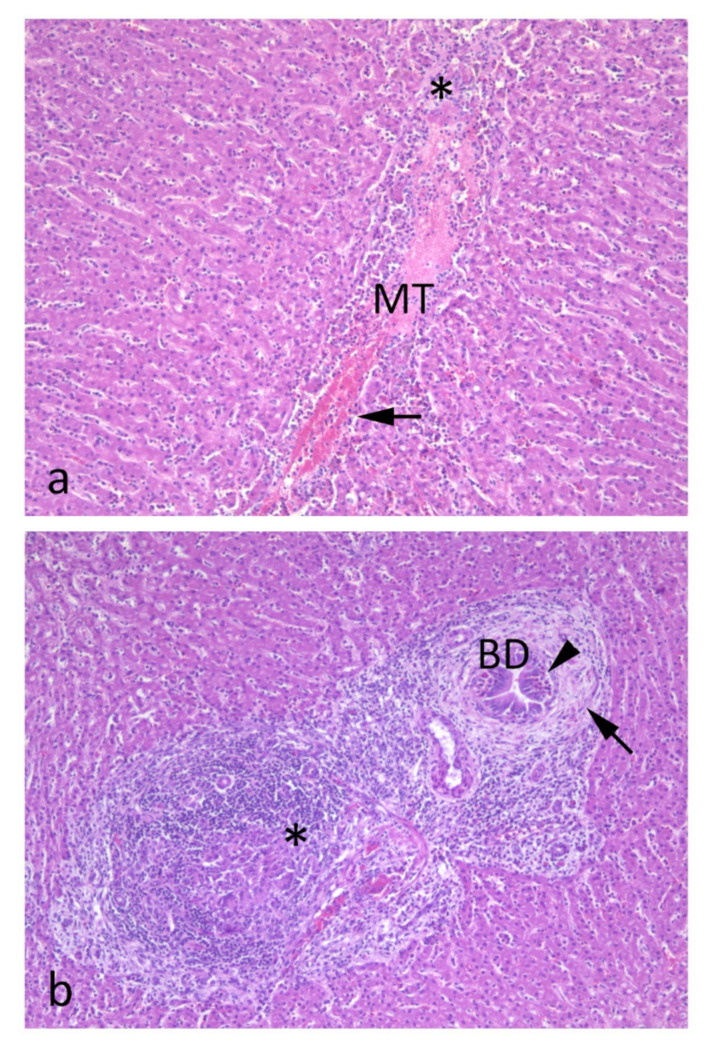
Histopathological changes of subacute fasciolosis. (**a**) Centrally placed is a migratory tunnel (MT) with destruction of the parenchyma, hemorrhage (arrow), inflammatory cells and fibrin (asterisk). (**b**) View of a triad. The bile duct (BD) is surrounded by connective tissue (arrow) and towards the duct epithelium there are eosinophils (arrowhead). The asterisk marks an area with giant cells and infiltration of mononuclear cells. There is also evidence of moderate bile duct hyperplasia.

Acute and sub-acute migration tunnels are evident as hemorrhagic areas with fibrin and degenerate hepatocytes, usually 2–3 mm in diameter (Figure 2a) [23]. Eosinophils will invade these areas early. Necrotic and degenerate tissue will be removed by macrophages and giant cells (Figure 2b) and replaced by granulation tissue rich in lymphocytes and eosinophils. In chronic cases, portal fibrosis with bile duct hyperplasia and infiltration of mononuclear cells and eosinophils will be evident (Figure 2b).

## 7. Diagnosis

The diagnosis in live animals is based on fecal examination, clinical presentation, hematology, serology, and molecular methods. However, all the diagnostic tests have limitations, especially for detecting early infection.

Coproscopy (flotation technique) is commonly used in diagnostics. The test is specific for detecting current infections, although challenging to use when the parasite burden is low. In addition, coproscopy does not detect non-reproducing immature stages and may also be unsuitable for large-scale and flock level testing [24].

Serological techniques increase diagnostic sensitivity, and several ELISA tests are commercially available [19,24]. Antibodies against *F. hepatica* can be detected 2–4 weeks after ingestion of metacercariae [25,26]. However, the test may be unable to differentiate between current and previous infections, since antibodies may persist for months after elimination of the parasite [27]. Maternal antibodies in lambs can last for up to 12 weeks ([14], Stuen personal communication). Serological tests should therefore not be used during the first two months on pasture. In addition, there seems to be a lack of correlation between antibody titer and the fluke burden [28].

Molecular methods are available for *F. hepatica* detection in fecal samples, such as PCR, loop-mediated isothermal amplification (LAMP) and recombinase polymerase amplification (RPA). A nested-PCR based on fecal samples may detect the infection at two weeks post infection, while with the LAMP method, the detection time can be as early as one week post infection [29,30]. RPA may be used in areas with limited resources since it does not involve thermal cycler equipment or is dependent on trained personnel [31]. However, further improvements and developments of the DNA-extraction methods are required to provide more reliable tests [32,33].

Meat inspection data can be used to diagnose *F. hepatica* infection, especially for slaughtered lambs, and can aid in early diagnosis before clinical signs of disease ([34], Ersdal personal communication). However, this method may lack sensitivity [35].

## 8. Treatment and Resistance towards Flukicides

Treatment with flukicides is often the only option to control *F. hepatica* infection in sheep. The ultimate aim is to use the right flukicide and dose at the right time and for the right reason in order to achieve fluke control. However, determining treatment time is challenging, since this depends on several factors, such as pasture, grazing period, infected snails, housing time and local climate.

Triclabendazole (TCBZ) is considered the drug of choice against *F. hepatica* due to its high efficacy against both the juvenile and adult stages. However, the continued use of TCBZ is threatened by multiple reports of resistant *F. hepatica*. TCBZ resistance has for instance been reported in sheep in several countries in Europe [11,36]. Other flukicides only active against adults include: albendazole, clorsulon, closantel, oxyclosantel, nitroxynil and rafoxanide [11,36]. However, resistance has already been reported for some of these [30,37,38,39,40]. To delay the onset of resistance, flukicidal drugs should be used strategically. Unfortunately, the traditional repeated blanket treatment regime has led to widespread resistance, not just to TBCZ, but also to several other flukicides [41]. Sheep do not develop functional immunity to *F. hepatica* and reinfection is therefore likely [11], and there is no evidence for natural innate immunity.

In case of TCBZ resistance, other flukicides such as nitroxinil and closantel may still have retained their effect in some areas [42]. However, a lack of flukicide efficacy does not necessarily mean resistance, since underdosing, inadequate storage of the anthelmintic, improperly applied anthelmintic, time of treatment or inappropriate diagnostic tests may be misinterpreted as anthelmintic resistance [43].

At present, there is no standard recognized protocol for the determination of efficacy or resistance [11]. The fecal egg count reduction test is still considered as the gold standard for the detection of anthelmintic resistance in *F. hepatica*, although it has several limitations because of intermittent shedding and release of eggs from the gall bladder [44,45]. Other methods to detect resistance include coproantigen ELISA and the *Fasciola* egg hatch test (FEHT) [45,46,47]. Due to the difficulties in detecting resistance based only on field trials, it has been suggested that field drug trials be combined with an in vitro test such as FEHT in order to verify resistance [43].

Quarantine treatment should be implemented for all purchased animals to avoid pasture contamination. If signs of fasciolosis persist following treatment, fecal examination should be performed three weeks after treatment. If eggs are still present, affected animals may be treated with an alternative flukicide, if possible [7]. There is also a growing concern related to drug residues in food and the impact they may have on the local environment [48].

No vaccine is currently available. Immune suppression and modulation by *F. hepatica* prevent the development of protective immunity by the host and have for decades challenged the efficiency of a potential vaccine. However, recent vaccine developments through genomic, transcriptomic and proteomic approaches are promising [49,50,51], in which the goal is to identify key molecules and antigen/adjuvant combinations that stimulate an adequate level of protective immunity [41,50,52]. The whole genome sequence of *F. hepatica* will hopefully accelerate this development [53].

## 9. Pasture Management

In areas where fluke infection is endemic, flukicidal drugs should be used in combination with grazing strategies. Grazing management is important to reduce exposure to liver flukes on pasture as well as the spread of anthelmintic resistance, and seems to be the most crucial factor for reducing the risk of *Fasciola* infection [34]. A fluke control management plan should be implemented, including investigation of all cases of sudden death, ill-thrift and weight loss in the actual flock. Fluke egg counts should be performed regularly. Data from abattoirs concerning liver condemnation due to liver flukes should be made available in order to monitor the occurrence of fluke infections. Risk pasture and risk periods should be identified based on annual rainfall, humidity, soil type and moisture, flock size, grazing period, availability of fresh grass, and other potential hosts (such as cattle or cervids) using the same grazing areas [54]. Climates with a high temperature and heavy rainfall have an important effect on the epidemiology of *F. hepatica* and may increase the risk of infection. Ongoing climate change may therefore extend the fluke transmission season by up to four months in northern Europe [55].

Fencing off or draining high-risk areas may be an alternative, although it may be difficult to identify snail habitats, as they can be localized and temporary [11]. Sheep should, if possible, avoid grazing snail-infected pastures in high-risk periods.

In some countries, weather forecasting systems have for years been available to help farmers decide when to treat [14,56]. Wet weather leading to standing water during the time of year when the temperature is above 10 °C but below 30 °C seems to be the key feature for identifying high-risk periods. However, drought conditions can lead to higher infection levels, as livestock may congregate around remaining drinking and grazing areas [11]. Meteorological (temperature, evaporation, rainfall) and soil data combined with laboratory (FEC, necropsy, serology, DNA methods) and slaughterhouse data should be used in future liver fluke forecast. Investigations of the environmental DNA of snails and *F. hepatica* may also be a tool for risk assessment and fluke control [57].

Another option may be reducing the population of infected snails in wet areas. Drainage and closure of open ditches are long-term methods to eradicate snail habitats permanently, but these may not be practical. Since several species, including cattle and wild species such as deer, rabbits and hares, can be infected with *F. hepatica* and function as reservoir hosts, it may be difficult to reduce the infection pressure in sheep flocks on infected pastures. In addition, *G. truncatula* may arrive from surrounding areas; a migration distance of up to 30 m has been observed [27]. Snail control using molluscicides should not be used due to adverse environmental effects.

On the basis of recent studies, it seems that there is a genetic and breed predisposition in susceptibility to liver fluke infection. However, potential breed and genetic differences should be elaborated more in the future [42].

## 10. Conclusions

*Fasciola hepatica* is an important trematode of domestic ruminants in areas where the environment is suitable for the intermediate host and causes a substantial economic loss due to death, wasting disease, production losses and the cost of treatment and prophylaxis. Grazing management and treatment with flukicide are, at the moment, the only options to control *F. hepatica* infection. A control management plan should be implemented in relevant areas, including investigations of all cases of death, ill-thrift and chronic wasting conditions. Risk pastures should be identified, reduced and avoided based on weather forecasts or historical data. Strategical flukicide treatment should be implemented to remove both immature and adult flukes.

Challenges related to fasciolosis in sheep will most probably increase and spread in the years to come due to climate change. Milder temperatures, heavier rainfall and a longer grazing period will improve the persistence of both the parasite and its intermediate hosts. The development of an accurate, rapid and cost-effective diagnostic test will hopefully allow farmers to treat animals earlier and in a more efficient and targeted manner in the future, and delay the spread of flukicide resistance. A commercial vaccine will also hopefully be available to increase the health and welfare of sheep on fluke-infested pasture.
